# An Efficient Method of Sharing Mass Spatio-Temporal Trajectory Data Based on Cloudera Impala for Traffic Distribution Mapping in an Urban City

**DOI:** 10.3390/s16111813

**Published:** 2016-10-29

**Authors:** Lianjie Zhou, Nengcheng Chen, Sai Yuan, Zeqiang Chen

**Affiliations:** 1 State Key Laboratory for Information Engineering in Surveying, Mapping and Remote Sensing, Wuhan University, 129 Luoyu Road, Wuhan 430079, China; zlj0808@whu.edu.cn (L.Z.); 2015206190004@whu.edu.cn (S.Y.); czq0119@whu.edu.cn (Z.C.); 2 Collaborative Innovation Center of Geospatial Technology, 129 Luoyu Road, Wuhan 430079, China

**Keywords:** cloud computing, data retrieval, Beidou positioning sensor, spatial-temporal data

## Abstract

The efficient sharing of spatio-temporal trajectory data is important to understand traffic congestion in mass data. However, the data volumes of bus networks in urban cities are growing rapidly, reaching daily volumes of one hundred million datapoints. Accessing and retrieving mass spatio-temporal trajectory data in any field is hard and inefficient due to limited computational capabilities and incomplete data organization mechanisms. Therefore, we propose an optimized and efficient spatio-temporal trajectory data retrieval method based on the Cloudera Impala query engine, called ESTRI, to enhance the efficiency of mass data sharing. As an excellent query tool for mass data, Impala can be applied for mass spatio-temporal trajectory data sharing. In ESTRI we extend the spatio-temporal trajectory data retrieval function of Impala and design a suitable data partitioning method. In our experiments, the Taiyuan BeiDou (BD) bus network is selected, containing 2300 buses with BD positioning sensors, producing 20 million records every day, resulting in two difficulties as described in the Introduction section. In addition, ESTRI and MongoDB are applied in experiments. The experiments show that ESTRI achieves the most efficient data retrieval compared to retrieval using MongoDB for data volumes of fifty million, one hundred million, one hundred and fifty million, and two hundred million. The performance of ESTRI is approximately seven times higher than that of MongoDB. The experiments show that ESTRI is an effective method for retrieving mass spatio-temporal trajectory data. Finally, bus distribution mapping in Taiyuan city is achieved, describing the buses density in different regions at different times throughout the day, which can be applied in future studies of transport, such as traffic scheduling, traffic planning and traffic behavior management in intelligent public transportation systems.

## 1. Introduction

The number of city vehicles, including buses, private cars, bicycles and electric vehicles, has increased exponentially [1,2]. In near real-time traffic, the time consumption of data retrieval is essential in applications such as pedestrian flow detection, urban traffic congestion and other near real-time applications. With so many vehicles, monitoring real-time operations is both necessary and essential [3]. Buses are one of the most common types of vehicle found in urban cities. To monitor their operational state, sensors are installed on buses, including speed-measuring instruments, positioning devices and other devices. The daily data volume of such sensor data is huge, reaching the scale of one hundred million [4]. The term “mass data” is defined as a collection of data sets whose volume and complexity make data management and processing using traditional tools difficult [5,6,7].

Currently, there are many vehicle-monitoring networks operated by different organizations in different urban cities. Table 1 lists several typical vehicle-monitoring networks, including the Shenzhen taxi network, Wuhan taxi network, Taiyuan bus network and New York taxi network. The volume of the data set generated each day by the Shenzhen taxi network is approximately 800 million items. Additionally, the data volume of the Wuhan taxi network is approximately 850 million, the data volume of the Taiyuan bus network is approximately 12.67 million and the data volume of the New York taxi network is approximately 984 million. Sharing records with specific temporal and spatial ranges from mass data sets is difficult and time consuming. Spatial and temporal mass data are defined as mass data containing spatial and temporal information. The characteristics of spatial and temporal mass data include large volumes, varieties, velocities and openness, as well as inappropriate structures and visualization difficulties, among others [8,9,10]. Mass spatio-temporal trajectory data has a similar definition as spatio-temporal mass data. In addition, mass spatio-temporal trajectory data sharing refers to mass spatio-temporal trajectory data retrieval and applications in our study.

Mass data sharing is difficult [11,12,13]. The bottleneck of data retrieval is the spatio-temporal index design, which promotes the efficiency of data retrieval. A spatial index significantly improves Big Earth Observation metadata retrieval performance by leveraging spatial relationships among features [14]. The R-tree [15] is one of the most popular spatial indices. Optimizations have been conducted using the R-tree and resulted in R-tree variants, such as the R+ tree [16], R* tree [17], Hilbert R tree [18], and APR-tree [19]. The R tree family is widely applied in spatial databases such as Oracle Spatial, PostgreSQL, and others [20,21]. NoSQL [22] is one of the most popular and novel databases designed for distributed management of big, unstructured or non-relational data. HBase [23] is one of the most popular NoSQL databases. SQLstream, a Big Data analysis tool, adopts Structured Query Language (SQL) for more reliable and simple querying. The other famous NoSQL databases are Apache Cassandra [24], Google BigTable [25], SimpleDB [26] and so on. Liakos [27] exploited this popular use of Extensible Markup Language and presented the means for querying metadata emanating from multiple sources in a succinct and effective way. Sun [28] proposed an incremental spreading activation algorithm to search for different types of information nodes gradually, promoting the associative retrieval of spatial Big Data. Storing large volumes of data is possible, but retrieving mass data based on the present data retrieval techniques is difficult. There are two problems in mass data sharing: (1) the inability to support quick data retrieval in near real-time traffic environments due to limited computational capability. The indexing and NoSQL database techniques can’t meet the data query efficiency for near real-time data retrieval as indicated in experiments. The data retrieval for MongoDB is about 10 s for one hundred million records. 10 s cannot meet the efficiency needs of real-time scenarios; (2) it is hard to meet mass data volume demands. Indexing and NoSQL have data record storage quantity limits, which are about one billion according to the references above. The size of the Taiyuan buses network data can reach billions, so a problem exists in the selected scenario. Impala, which is developed by the Cloudera Company, has been applied in high-performance data retrieval [29]. Impala is a great tool, drawing on the idea of mass parallel processing using a parallel database by applying the SQL query paradigm. As the experiments described in Section 3 indicate, we propose an efficient spatio-temporal trajectory data retrieval method (ESTRI) based on Cloudera Impala to enhance the efficiency of mass data sharing. By extending the data retrieval interface of Impala, the study achieves efficient data retrieval of spatio-temporal trajectory data. In addition, our study is designed to solve the mass trajectory data retrieval difficulty. The Taiyuan BD buses network was selected as the experimental network. As the data size in Taiyuan buses network can reach 20 million each day, so it’s difficult to retrieve the wanted data in the mass data of a period such as one month, which reaches 600 million records. Compared with the existing methods, ESTRI can achieve efficient data retrieval, as shown in experiments. As the data retrieval is an important step in data sharing and Hadoop Distributed File System (HDFS) is an efficient storage center, so ESTRI can achieve efficient data sharing for mass data and provide a possible solution to this kind of problem.

This paper is organized as follows: we describe the Impala-enabled spatio-temporal trajectory data sharing methodology in Section 2, where the algorithm of the proposed method is also described. Experiments based on bus data are performed and the performance of the proposed method is evaluated in Section 3. Section 4 provides a discussion, and the conclusions of the study and potential future directions are discussed as well.

## 2. Impala-Enabled Spatio-Temporal Mass Data Sharing Methodology

The proposed methodology is presented in Section 2. Section 2.1 describes the mechanism of Cloudera Impala and core parts; Section 2.2 describes the algorithm of spatio-temporal trajectory data retrieval; Section 2.3 describes the data block division of data in distributed environments; Section 2.4 describes the data tables for storing trajectory data and metadata for distributed environment.

### 2.1. Mechanism of Impala and Core Parts

**Figure 1:**
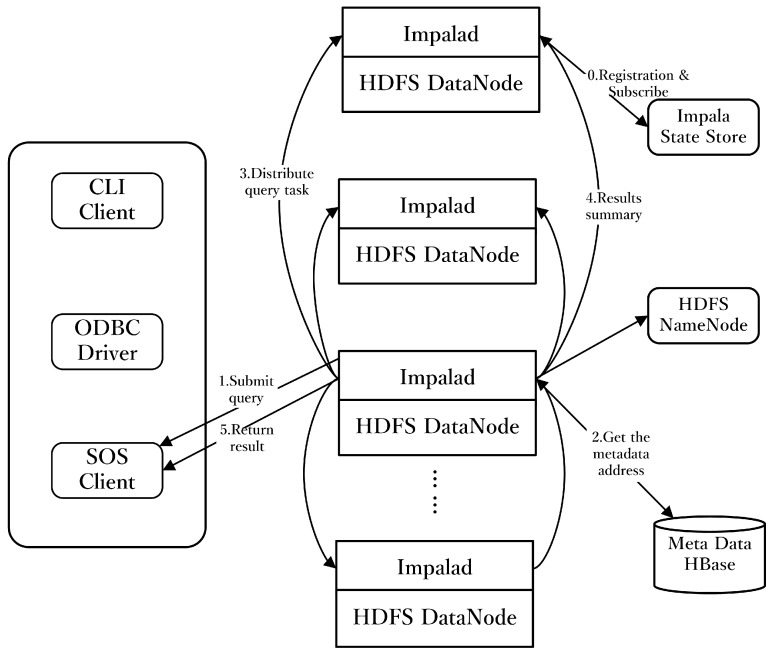
Impala query processing diagram.

Widely applied by companies such as Twitter, Impala is a suitable tool for data retrieval in mass data environments [30]. As stated by the Cloudera Company, Impala can achieve an efficiency 3 to 90 times greater than that of Hive [31]. Data are stored in HDFS, instead of in a database such as PostgreSQL or MySQL. Impala mainly comprises three parts: Impalad, State Store and Command-line interface (CLI). The Impala query-processing diagram is shown in Figure 1. First, a user submits the SQL statement to one of the Impalad nodes using the CLI, an Open Database Connectivity (ODBC) driver or a Sensor Observation Service (SOS) client. Then, the query planner of the node parses the SQL statement and generates an execution plan. Second, having performed the initialization, Impala assigns the execution plan to multiple Impala query executors at other nodes. Finally, the execution plan of Impala is executed in parallel using each node’s Random Access Memory (RAM). The intermediate results will be transmitted from each Impalad process in stream mode. The final results will be returned to the client through streaming data.

### 2.2. Spatio-Temporal Trajectory Data Sharing Algorithm Enabled by Impala

The data retrieval algorithm is designed to share the data stored in HDFS with Impala. As Figure 1 shows, the input can be from a CLI client, ODBC client or SOS client. The location of the data block should be retrieved according to the spatio-temporal trajectory data sharing algorithm. Impala supports customized query statements in data retrieval. Therefore, a query statement should be constructed based on the query rules of Impala. The data are retrieved by judging whether the data are in the spatio-temporal range. By inheriting the customized algorithm, it can execute the designed function and determine if the data are in the spatio-temporal query range using the *SpatialPointInOrNot*(*P_longitude_*, *P_latitude_*, *Point_tn_m_*) and *TemporalQueryBlock*(*P_begin_*, *P_end_*) functions. By traversing the data stored in *block_tn_spatial_*, the data in the specified spatio-temporal query range can be retrieved via the judgment. The algorithm of spatio-temporal trajectory data retrieval is shown in Algorithm 1. The input contains the *ST-Box* and *sensorID* information. Besides, the output is *ObservationCollection*, which contains the retrieved data and the metadata information. The involved HDFS data partitioning rule is described in Section 2.3 in detail.

**Algorithm 1.** Algorithm of spatio-temporal trajectory data retrieval  **Input**: current spatio-temporal data retrieval range *ST-Box*(*P_longitudeRange_, P_latitudeRange_, P_begin_, P_end_*)  **Output**: query results *ObservationCollection*  **Use**: *SensorObservationService*(*Data_tn_Input, AlgorithmID_IMPALA_, ResponseFormat*) inherits the data access object of *SOS* implementation    *doConfiguration*(*Path_HDFS_, Number_port_, URL_SOS_*) configures *Path_HDFS_*, *Number_port_* and *URL_SOS_*    *SpatialQueryBlock*(*P_longitudeRange_, P_latitudeRange_*) positions the spatial block according to the spatial range    *TemporalQueryBlock*(*P_begin_, P_end_*) positions the spatial block according to the spatial constraint    *SpatialPointInOrNot*(*P_longitude_, P_latitude_, Point_tn_m_*) judges whether the point is in the spatial block    *TemporalBlockInOrNot*(*P_begin_, P_end_, Point_tn_m_*) judges whether the point is in the spatial block  **STEP 1:** Inherit the mandatory interface in *SOS* implementation through the *SensorObservationService*(*Data_tn_Input, AlgorithmID_IMPALA_, ResponseFormat*) function in SOS implementation.  **STEP 2:** Start configuring the parameters of the input path of the HDFS’ IP address and port number of entry of the Impala cluster. Create a JDBC using the parameters configured above and the function *doConfiguration*(*Path_HDFS_, Number_port_, URL_SOS_*) to connect the Impala and HDFS layer.  **STEP 3:** Obtain the objects *ST_tn_* information from ST-Box using the *get4*(*ST-Box*) function, which is developed based on the *Observation & Measurement* encoding model. The spatio-temporal metadata are used to construct a spatio-temporal query statement and obtain the retrieved data from the HDFS.  **STEP 4:** Implement the function *SpatialQueryBlock*(*P_longitudeRange_, P_latitudeRange_*) to achieve the specified *block_tn_spatial_* acquisition. Via the function, the location of *block_tn_spatial_* in the HDFS can be obtained. The data located in the acquired *block_tn_spatial_* can be obtained from step 5 in detail.  **STEP 5:** Implement the function *SpatialPointInOrNot*(*P_longitude_, P_latitude_, Point_tn_m_*) to judge whether *Point_spatial_m_* is in the range between *P_longitude_* and *P_latitude_ or not*. By traversing all the points in *block_tn_spatial_*, the points meeting the judgement criteria are discovered. The function judges whether the point in *block_tn_spatial_* is in the specified spatial range.  **STEP 6:** Implement the function *TemporalQueryBlock*(*P_begin_, P_end_*), achieving the specified *block_tn_temporal_* discovery. Via the function, the id of the *block_tn_* can be discovered. The function judges whether the point in *block_tn_spatial_* is in the specified temporal range. The data located in the acquired *block_tn_temporal_* can be obtained from step 7 in detail.  **STEP 7:** Implement the function *TemporalBlockInOrNot*(*P_begin_, P_end_, Point_tn_m_*) to judge whether *Point_tn_m_* is in the range between *P_longitude_* and *P_latitude_*. By traversing all the points in *block_tn_temporal_*, the points meeting the judgement criteria are discovered. Package the data in *Point_spatial_m_* and *Point_temporal_m_* to assemble the *obsevationCollection* and return it to the SOS or CLI.

The extension of Impala to support spatio-temporal data retrieval is essential in spatio-temporal trajectory data retrieval process. Impala provides the interface “udf-spatial” for user-defined query operation. By overwriting *SpatialPointInOrNot*(*P_longitude_*, *P_latitude_*, *Point_tn_m_*) and *TemporalQueryBlock*(*P_begin_*, *P_end_*) function, the designed spatio-temporal trajectory data retrieval function can be implemented.

### 2.3. Block Division for Indexing the Spatio-Temporal Trajectory Data

Data partition can help divide the data blocks into multiple blocks based on the geographical position of the stored data in HDFS. Similar to data indexes in relational database, the block partitions can speed up data retrieval. The rules of blocks division is based on longitude grid and time block. The longitude grid is organized by the latitude and longitude. The data size of standard data block is 20 megabyte, so the number of data blocks can be calculated via Equation (1):

(1) $$N_{Block}=\frac{RAM_{o}\times N}{10}$$

where *N_Block_* refers to the number of data blocks; *RAM_o_* refers to the RAM of single node; *N* refers to the number of the nodes in the cluster. Analyzing the mass data in HDFS, the latitude and longitude grid can be applied to construct the blocks division for indexing the spatio-temporal data. Adopting a Hilbert encoding pattern, the data stored in HDFS can be encoded by the designated encoding pattern. However, the data should be divided into numerous blocks to promote the data retrieval efficiency. In Impala, the data is divided according to the average distribution rule. Obtaining the minimum or maximum value of the longitude and latitude of the spatio-temporal data in HDFS, the average degree of block can be calculated according to Equation (2):

(2) $$Degree_{mean}=\sqrt{\frac{(Longitude_{max}-Longitude_{min})\times \left(Latitude_{max}-Latitude_{min}\right)}{N_{Block}}}$$

Here the parameter *Longitude_max_* refers to the max value of the longitude; the parameter *Longitude_min_* refers to the min value of the longitude; the parameter *Latitude_max_* refers to the max value of the latitude; the parameter *Latitude_min_* refers to the min value of the latitude and the parameter *Degree_mean_* refers to the degree of partition interval.

**Figure 2:**
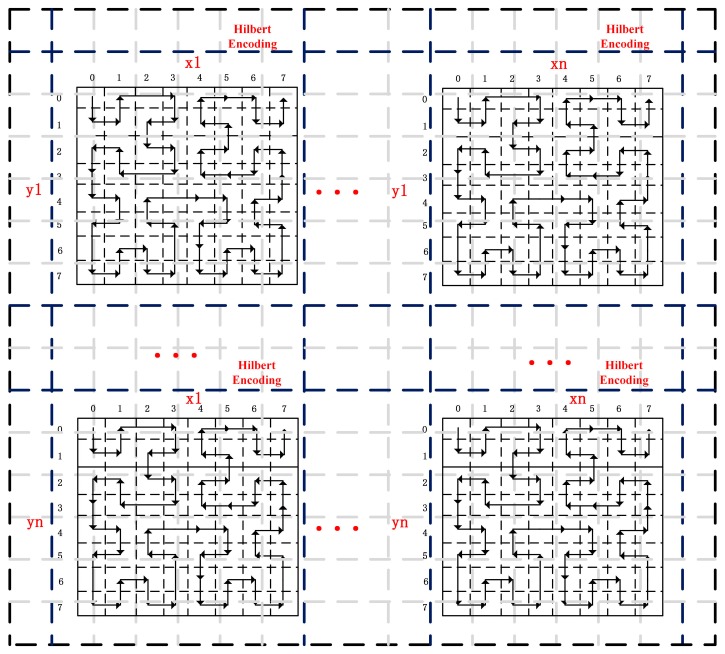
Hilbert encoding process in the HDFS data blocks.

Hilbert curve encoding [32] is a classic indexing method to encode adjacent geographical objects. The Hilbert curve can effectively map one-dimensional curves in two dimensions, maintaining the topological properties of the data. Hence, Figure 2 shows the Hilbert encoding process in the HDFS data blocks. The red dotted lines stand for single encoding blocks, and the black solid lines stand for boundaries of parquet data blocks in the HDFS. In addition, horizontally or vertically, there are n zones in Hilbert encoding. In each encoding zone, there are a certain number of data blocks. In data retrieval process, the data blocks containing data to be retrieved are obtained first, and then the data in each block are retrieved.

The data in the HDFS of Impala are stored with parquet. Parquet is a column-oriented binary file format intended to be highly efficient for large-scale queries. In addition, parquet is especially good for queries that require scanning of particular columns within a table, e.g., querying “wide” tables with many columns, or performing aggregation operations such as SUM() and AVG() that need to process most or all of the values in a column. The parquet tables are stored in RAM; thus, the input/output of parquet tables is fast.

### 2.4. Approach for Integrating Impala and SOS

To integrate Impala with SOS, there are some considerations. First, the SOS data access abstract class should be inherited, and the implementation should be overwritten. However, the HDFS supports none spatio-temporal query. Therefore, the spatio-temporal query function should be developed based on the user defined function. Second, HDFS provides the capability of data storage. Hence, integrating Impala with SOS should focus on the improvement of Impala implementation. Impala was developed in the C++ programming language and the Open Source Geospatial Foundation (OSGeo) third-party library, which is a C++ port of the Java Topology Suite. The suite includes spatial predicate functions and spatial operators, as well as specific java topology suite enhanced topology functions [33]. Hence, the OSGeo library can be applied, and the self-defined function of the spatio-temporal query must be developed based on the OSGeo library.

**Figure 3:**
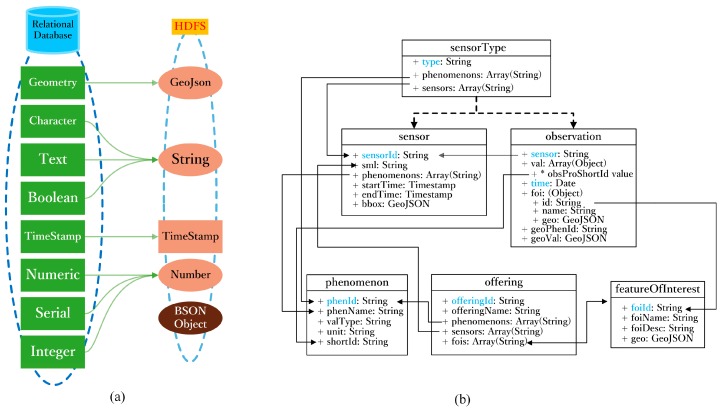
(**a**) The mapping of the data types from a relational database to the HDFS; (**b**) Tables design in HDFS for the SOS.

The SOS [34,35,36] offers pull-based access to sensor measurements or metadata and provides standardized access to sensor observations and sensor metadata [37,38]. Therefore, SOS provides the capability of accessing spatio-temporal trajectory data. Open Geospatial Consortium (OGC) provides the interface of the Observation & Measurements [39,40], which encodes the data and metadata associated with observations. Similar to Observation & Measurements, the tables in the HDFS of the SOS comprise most of the metadata associated with Taiyuan BD bus observations. Figure 3a describes the mapping of the data types from a relational database to the HDFS. The arrow in Figure 3a shows the relationship between different tables. In HDFS, those tables can then be accessed with standard SQL syntax. Impala supports most of the SQL-92 SELECT statement syntax, as well as additional SQL-2003 analytical functions and most of the standard scalar data types, including integer, floating point, STRING, CHAR, VARCHAR, TIMESTAMP, and DECIMAL, with up to 38 digits of precision. Figure 3b describes the tables designed in the HDFS for the SOS. Different from the relational database, the geometry data type is converted to GeoJson. A GeoJson object can represent the geometry, feature or feature set in the common encoding format of JavaScript object notation.

The designs of the tables can be summarized as follows:

(1) sensorType table: Stores the sensor type information of the sensors and phenomenon.
(2) sensor table: Stores the sensor information, including the sensor metadata, the time when the sensor begins observation and the time when the sensor ends observation.
(3) observation table: Stores the observation information when the sensors finishes observation, including the observation time, spatial range of the observation and the observation result.
(4) phenomenon table: Stores the phenomenon information, including the phenomenon name, observation value type and unit information.
(5) offering table: Stores the organization information, including the organization name, phenomenon name and sensor information.
(6) featureOfInterest table: Stores the spatial arrangement of observations, including the name and coordinate encoded within the GeoJson data type.

## 3. Experiments and Discussion

### 3.1. BD Bus Network and Experimental Environment

**Figure 4:**
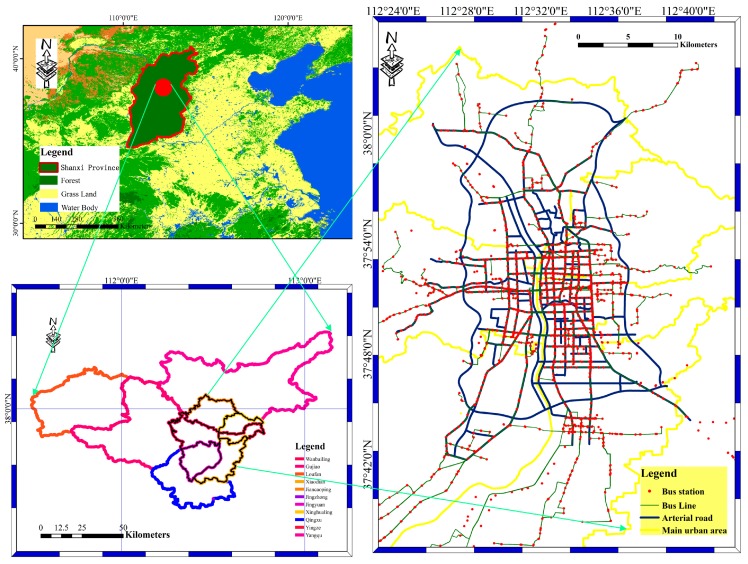
Spatial distributions of bus stations, bus lines and arterial roads in Taiyuan, Shanxi Province, China.

Our study chooses experimental data sets from the Taiyuan BD bus network, containing 2300 BD buses. Every 20 s, observed data, which consists of vehicle speed information, vehicle location information, and observation time information, are recorded. In addition, the number of records are approximately 20 million each day. Figure 4 shows the spatial distributions of bus stations, bus lines and arterial roads in Taiyuan city, Shanxi Province, China. In the right subfigure, red points stand for bus stops, yellow lines stand for bus lines, and blue lines stand for arterial roads in Taiyuan city. There are six districts and three countries in Taiyuan city. There are approximately 800 bus stations, 400 bus lines, and 100 arterial roads in the city, with approximately 2300 BD buses running on the 400 bus lines. The TAX408BD sensor fixed on Taiyuan buses is a module with a small volume, high sensitivity, and low power consumption that is easy to integrate. Widely used in the fields of shipping, road traffic, vehicle monitoring, vehicle navigation, handheld tracking and goods tracking, the bus sensor has features such as high precision of real-time, three-dimensional positioning, three-dimensional velocity, and timing capability. After the GPS of USA and the Global Navigation Satellite System satellite navigation system of Russian, the Chinese BD satellite navigation system is the third oldest satellite navigation system in the world. In addition, the positioning accuracy of the China BD satellite navigation system is generally equal to that of GPS [41].

The hardware environment is designed in a distributed form. We deploy the Impala implementation on five computers with the same configuration, i.e., identical operating system (CentOS 7.0-1406) and Cloudera Impala 2.0 are installed on each node. The configuration of each node is i7 4720HQ (6 M cache, 8 Cores, 2.60 GHz, 5 GT/s) with 8 gigabyte RAM. They are connected to 40 GB/s InfiniBand. The Impala environment contains four Datanodes and one Namenode.

The 52° North SOS [42,43] is applied in the experiment. The 52° North SOS is improved or adjusted to support access to the persistent layer of the HDFS. The SOS provides a data access layer through a Data Access Object paradigm. Three overwritten Data Access Object classes contains: GetCapabilityDAO, DescribeSensorDAO, and GetObservationDAO. GetObservationDAO can be inherited to provide the capability of accessing observations in the persistent layer. Because Impala has been closely integrated with HDFS, so a user can access the Impala web address http://localhost:21050 to further understand the cluster operation situation. The Impala cluster provides strong processing and storage capabilities, and the resource consumption of the cluster is displayed in real time via the main page.

### 3.2. Data Partitions and Data Encoding

To promote the data retrieval efficiency, more data blocks work. However, the size of a data block is difficult to set up. Too many blocks will occupy too much memory, potentially exceeding the memory limit of the machine. The partition mechanism of BD spatio-temporal trajectory data in the HDFS is described in Section 2.3; therefore, the number of the block partitions can be calculated. In addition, the resource consumption mechanism of Impala occupies memory and improves the data retrieval efficiency; thus, more block partitions would require more memory. The number of block partitions *N_Block_* is the specified amount as described in Equation (1), which is 4096. Considering the latitude and longitude range, the *Degree_mean_* is 0.0063° according to Equation (2). Therefore, the consumed memory of each node in the cluster will not exceed the maximum machine memory limit, reaching approximately 8 gigabyte in the cluster.

For example, an encoding of the blocks is “2010 1833 1830 1829” in the experiment. After encoding the blocks with the Hilbert curve, the data blocks can be queried in a short time based on the encoding mechanism. By reversing the specific encoding value to obtain the binary value of the encoding number, the rank and row can be calculated according to the encoding rules in Section 2.3. Besides, Impala supports fast data access, as shown in the following sections, based on its data partition mechanism and memory mechanism. Consequently, the data in HDFS blocks can be retrieved effectively and efficiently.

### 3.3. Data Retrieval at Different Times

The input of data retrieval with ESTRI is the request in SOS or CLI. The output of data retrieval is the BD bus metadata that satisfies the spatio-temporal range. In SOS, the request is a standard request encoded with Extensible Markup Language. In CLI, the request is an SQL statement. BD bus metadata contain BD bus locations and metadata information, such as bus name, speed, position and location information. Some buses are located in the map view, while most buses are located on the main roads of Taiyuan city, such as Yingze Avenue, Gongyuan Avenue, Liuxiang Avenue and Wenmiao Avenue. The data are retrieved from the HDFS via Impala implementation. By sending a GetObservation request to the SOS server, the data stored in the HDFS can be retrieved and displayed on the map of Taiyuan city. Using bus “晋A94369” as an example, the location, position, speed and other information can be accessed from the HDFS (“lon”: “112.58497”, “lat”: “37.58712”, “suid”: “100”, “speed”: “0”, “vdesc”: “103路”). In addition, many buses travel on the main roads, resulting in a high bus density on those roads, while the bus density in residential communities or business zones is small or zero.

**Figure 5:**
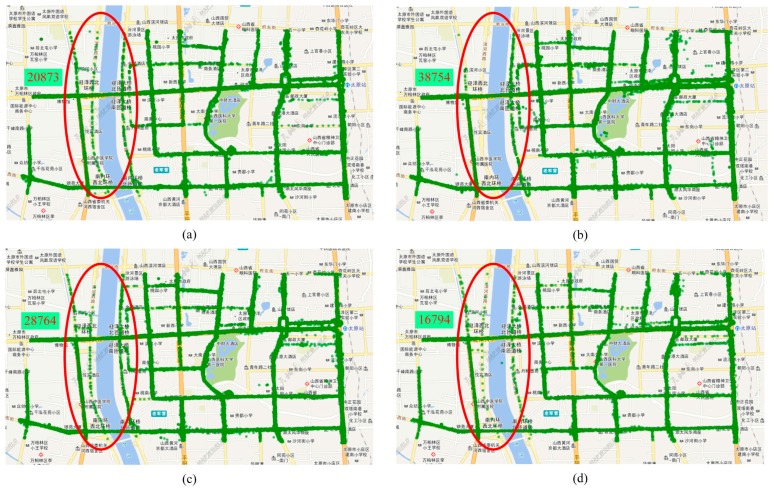
Retrieved BD bus locations in the Taiyuan national high tech Industrial Development Zone at different times: (**a**) 6:20 a.m. to 7:20 a.m.; (**b**) 11:20 a.m. to 00:20 p.m.; (**c**) 4:20 p.m. to 5:20 p.m.; and (**d**) 6:20 p.m. to 7:20 p.m.

Figure 5 shows the buses’ location in the Taiyuan national high tech industrial development zone at different times such as (a) 6:20 a.m.; (b) 7:20 a.m.; (c) 4:20 p.m.; and (d) 5:20 p.m. Green spots stand for the bus stops and yellow lines on the map stand for the main roads in Taiyuan city while the green spots cluster in the red circle from (a) to (d) changes severely. At different times, the density of the green spots is different in the same areas according to the four maps. The query latitude range is from North latitude 37.12° to 37.88° and the query longitude is from east longitude 112.13° to 112.63°. As shown in Figure 5, about 20873 buses in the red circle are retrieved from 6:20 a.m. to 7:20 a.m. in Figure 5a; about 38754 buses in the red circle are retrieved from 11:20 a.m. to 00:20 p.m. in Figure 5b; about 28764 buses in the red circle are retrieved from 4:20 p.m. to 5:20 p.m. in Figure 5c; and about 16794 buses in the red circle are retrieved from 6:20 p.m. to 7:20 p.m. in Figure 5d. The number of retrieved buses varies in one day. Moreover, the number of retrieved buses are greater during traffic peaks in one day.

**Figure 6:**
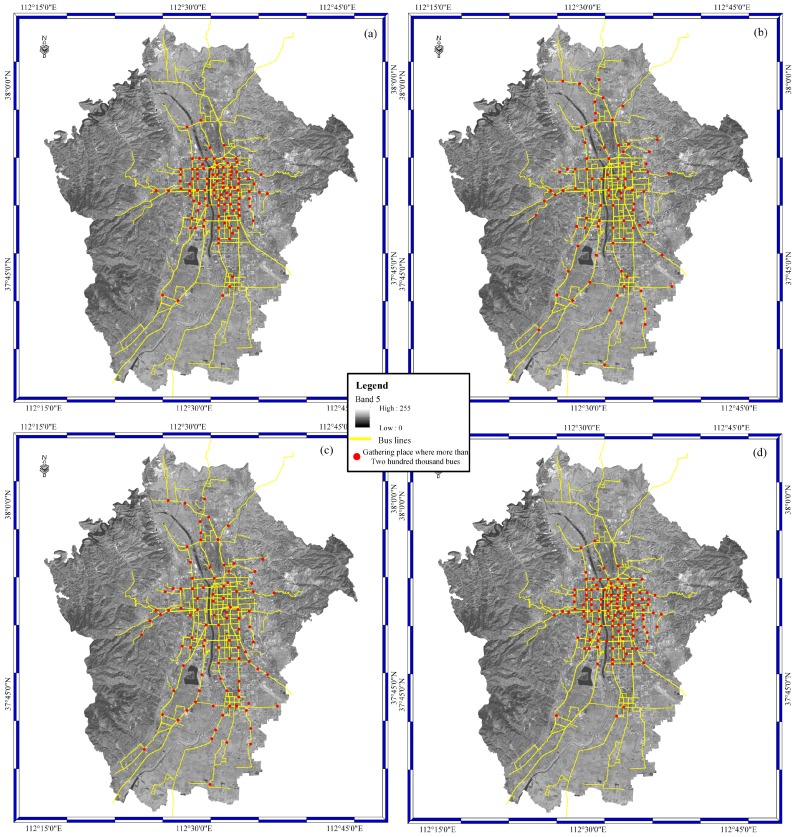
Buses distribution map of Taiyuan city: (**a**) from 7:30 a.m. to 9:00 a.m.; (**b**) from 11:00 a.m. to 12:00 a.m.; (**c**) from 12:30 p.m. to 2:00 p.m.; and (**d**) from 7:30 p.m. to 9:00 p.m.

Figure 6 shows a bus distribution map of Taiyuan city. The base map is the Landsat 8 Operational Land Imager image. Band 5 is applied to show the general outline of Taiyuan city. The red spots are areas where the number of buses is more than two hundred thousand, and the yellow lines stand for bus lines. From Figure 6a–d, the query time range is from 7:30 a.m. to 9:00 a.m. in Figure 6a, from 11:00 a.m. to 12:00 a.m. in Figure 6b, from 00:30 p.m. to 2:00 p.m. in Figure 6c, and from 7:30 p.m. to 9:00 p.m. in Figure 6d. As shown in Figure 6, the buses gather on the main roads in the urban district from 7:30 a.m. to 9:00 a.m. and 7:30 p.m. to 9:00 p.m. The buses gather on the main roads in urban and suburban areas from 11:00 a.m. to 12:00 a.m. and 00:30 p.m. to 2:00 p.m. Consequently, the data retrieval results suggest that buses are located in urban areas in Taiyuan city during rush hour in the morning and evening, and buses are located in urban and suburban areas in Taiyuan city during normal daytime working hours. Furthermore, congestion may occur during rush hour in the morning and evening, rather than during normal daytime working hours.

### 3.4. Performance Evaluation

To validate the efficiency of ESTRI, the experiment comparison between the ESTRI performance and existing methods is essential. The spatial range is the bounding box of (112.522 37.872 112.586 37.872 112.587 37.836 112.517 37.837 112.522 37.872) and the temporal range is from “2015-04-01 09:29:08” to “2015-04-01 12:01:09”; the vehicle ID is “urn:liesmars:mobilesensor:taiyuanbus:晋A81893”.

**Figure 7:**
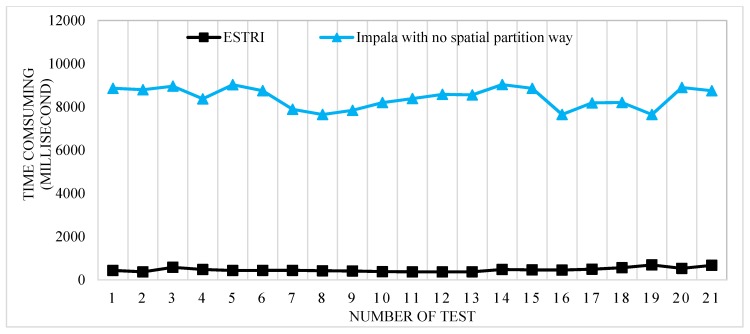
Time consumption for fifty hundred data records between ESTRI and Impala with no spatial partition way.

In the experiment, data whose volumes range from fifty million to two hundred million records are tested. According to the blocks dividing method in Section 2.2, the spatial area of the records is divided into 2406 data blocks, compressed with the parquet file format. Figure 7 shows the time consumption in fifty hundred is 5000 ms with ESTRI, spatial partition and no partition, spatial position way. When the data volume is fifty million, the mean record retrieval time consumption in ESTRI is 436 ms. In the no spatial partition way, the mean time consumption is 8863 ms. According to Figure 7, the time consumed by ESTRI is much lower than Impala with no spatial partition way. With data partition as proposed in Section 2.3, the efficiency of data access can be accelerated greatly. It is found that the ESTRI performs better compared with Impala with no spatial partition way, so the proposed spatial partition way of data in HDFS is effective.

**Figure 8:**
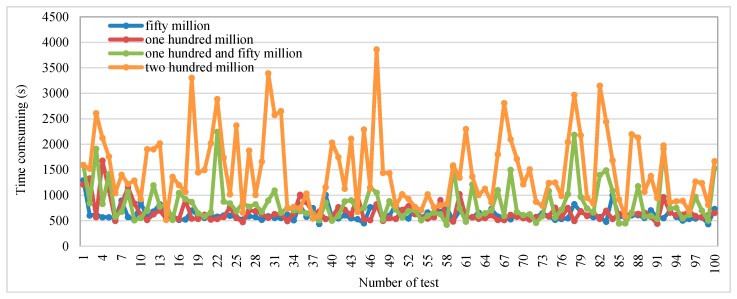
Time consumption in different data volume with ESTRI.

Figure 8 shows the time consumption for different data volumes with ESTRI. The blue line stands for time consumption with ESTRI at fifty million data volume; the red line stands for time consumption with ESTRI at one hundred million data volume; the green line stands for time consumption with ESTRI at one hundred and fifty million data volume; the yellow line stands for time consumption with ESTRI at two hundred million data volume. Overall, the time consumption with ESTRI at two hundred million data volume is higher than at other data volumes during 100 tests, as shown in Figure 8.

**Figure 9:**
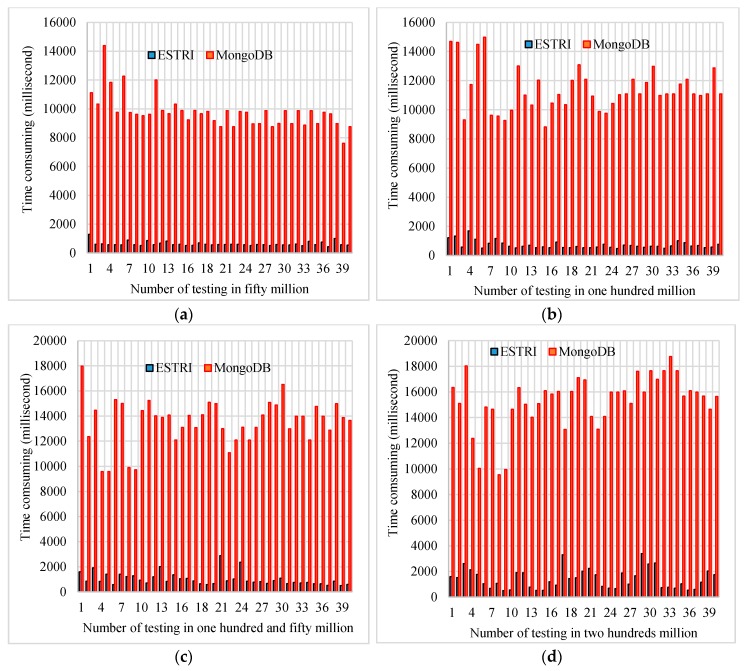
Time consumption comparisons between ESTRI (black color) and MongoDB (red color) for different amounts of data: (**a**) fifty million data; (**b**) one hundred million data; (**c**) one hundred and fifty million data; and (**d**) two hundred million data.

The consuming time comparisons between ESTRI and MongoDB [44] for different data volumes is shown in Figure 9. In fifty million, one hundred million, one hundred and fifty million and two hundred million data, the time consumption can be compared with that in ESTRI. At fifty million data volume, the mean time consumption of ESTRI is 603.3 ms, while the time consumption that MongoDB achieves is about 9247.3 ms; for one hundred million data, the time consumption of ESTRI is about 647.5 ms and the time consumption of MongoDB is about 11,262.4 ms; for a one hundred and fifty million data volume, the mean time consumption of ESTRI is about 943.5 ms and the time consumption of MongoDB is about 13605.75 ms; at two hundred million data volume, the mean time consumption of ESTRI is about 1432.3 ms and the time consumption of MongoDB is about 16019.6 ms. For fifty million data volume, one hundred million data volume, one hundred and fifty million data volume and two hundred million data volume, the time consumption of ESTRI is about seven times lower than that in MongoDB. The four subfigures reveal the data size can influence the data retrieval time consumption, so the data size is a key factor influencing the data retrieval time. Moreover, from fifty million to two hundred million record data size, the ESTRI data retrieval time consumption is much lower than that of MongoDB, so the efficiency of ESTRI is much higher than MongoDB for mass data.

To summarize, on the one hand, ESTRI achieves better efficiency than Impala with no spatial partition way. On the other hand, ESTRI can achieve efficient data retrieval at the one hundred million data volume scale compared to the retrieval efficiency of MongoDB. From data volumes of fifty million to two hundred million, the performance of ESTRI is approximately seven times better than MongoDB. Thus, for mass data volumes, especially at the one hundred million data volume scale, ESTRI is an efficient spatio-temporal trajectory data retrieval method for mass BD bus data due to distributed computation ability and the ESTRI algorithm proposed in Section 2.2.

## 4. Conclusions and Future Work

With mass data hard being to retrieve as described in the Introduction section, this study proposes ESTRI to enhance the capability of mass data sharing in near real-time application scenarios by extending the data retrieval interface. On one hand, the proposed ESTRI can promote the efficiency of mass data retrieval compared with Impala with no spatial partition as indicated in Section 3.4. On the other hand, as tested experimentally, ESTRI performs better compared with MongoDB. The performance of ESTRI is about seven times higher than that of MongoDB. The mean query time of ESTRI is approximately 1 s for a data volume of one million and 2 s for a data volume of two million. Moreover, the bus distribution was mapped from 7:30 a.m. to 9:00 a.m., 11:00 a.m. to 12:00 a.m., 12:30 p.m. to 2:00 p.m., and 7:30 p.m. to 9:00 p.m. Compared with other existing indexing techniques and NoSQL datastores, ESTRI can achieve efficient data retrieval, especially for mass data volumes such as one hundred million records, as indicated in the experiments. The time consumption of ESTRI with 200 million data size is about 2 s and the efficiency of ESTRI is about 7 times higher than that in MongoDB, which can meet the efficiency demands of quick data retrieval in near real-time traffic environments.

To put it into practical transport application, the ESTRI methodology should take the number of computers into account to balance the cost and the efficiency. The proposed methodology can be applied in future studies of transport, such as traffic scheduling, traffic planning and traffic behavior management in intelligent public transportation systems.

## Figures and Tables

**Table 1:** Heterogeneous and typical vehicle monitoring networks.

**Bus Network**	Shenzhen taxi network	Wuhan taxi network	Taiyuan bus network	New York taxi network
**Location**	Shenzhen, China	Wuhan, China	Taiyuan, China	New York, America
**Locating Device**	Global Position System (GPS)	GPS	BeiDou (BD) Navigation Satellite System	GPS
**Start Date**	February 2011	March 2012	August 2013	June 2009
**Vehicle Number**	25,000	12,137	2200	33,000
**Measurement Attribute**	Speed, location, direction	Speed, location, direction	Speed, location, direction	Speed, location, direction
**Observation Interval (Seconds)**	60	30	30	30
**Data Produced per Day**	800 million	860 million	12.67 million	984 million
**Data Storage**	Oracle	MongoDB cluster	MySQL	Unknown

## Abbreviations

The following abbreviations are used in this manuscript:

ESTRI	Efficient spatio-temporal data retrieval method based on Impala
RAM	Random Access Memory
NoSQL	Not Only SQL
CLI	Command-line interface
ODBC	Open Database Connectivity
SQL	Structured Query Language
HDFS	Hadoop Distributed File System
SOS	Sensor Observation Service
BD	BeiDou
GPS	Global Position System
OSGeo	Open Source Geospatial Foundation
OGC	Open GIS Consortium
WKT	Well-Known Text
GML	Geography Markup Language
